# Supplementary material for: Comorbidity trajectories before and after the diagnosis of heart failure: A UK Biobank cohort study
Source: Eur J Heart Fail. 2025 Feb 11;27(11):2259–69. doi: 10.1002/ejhf.3601 (PMC12765044; doi:10.1002/ejhf.3601)
Supplement: Supplementary file 1 — Appendix S1. Supporting Information. [file EJHF-27-2259-s001.docx]

**SUPPLEMENTARY MATERIAL**

**Comorbidity trajectories before and after the diagnosis of heart failure: a UK Biobank cohort study**

Hugo MacGowan^1^*, Oliver I Brown^1^*, Michael Drozd^1^, Andrew MN Walker^1^, Marilena Giannoudi^1^, Sam Straw^1^, Maria F Paton^1^, John Gierula^1^, Melanie McGinlay^1^, Kathryn J Griffin^1^, Klaus K Witte^1^, Mark T Kearney^1^, Richard M Cubbon^1^

^1^ Leeds Institute of Cardiovascular and Metabolic Medicine, University of Leeds, UK

* Denotes joint first authorship

**Corresponding author:**

Dr Richard Cubbon. LIGHT Laboratories 7.04, University of Leeds, Clarendon Way, Leeds, LS2 9JT, United Kingdom

Tel: +44 3430785

Email: r.cubbon@leeds.ac.uk

**Contents**

Page 3 Supplemental Table 1: UK Biobank definitions of HF and comorbidities.

Page 5 Supplemental Table 2: Timing of comorbidity diagnoses in relation to HF diagnosis in participants with at least 5 years follow-up or death in this period.

Page 6 Supplemental Table 3: Timing of comorbidity diagnoses in relation to HF diagnosis stratified by sex.

Page 7 Supplemental Table 4: Timing of comorbidity diagnoses in relation to HF diagnosis stratified by age at HF diagnosis.

Page 9 Supplemental Figure 1: Timing of comorbidity diagnoses in relation to HF diagnosis stratified by sex.

Page 11 Supplemental Figure 2: Timing of comorbidity diagnoses in relation to HF diagnosis stratified by age at HF diagnosis.

**Supplemental Table 1: UK Biobank definitions of HF and comorbidities**

| **Characteristic** | **Definition Source** |
| --- | --- |
| Anaemia | Field IDs 130622, 130623, 130624, 130625, 130626, 130627, 130628, 130629, 130648, 130649, 130642,130643, 130640, 130641, 130636, 130637, 130638, 130639, 130634, 130635 |
| Asthma | Field IDs 42014, 42015 |
| Atrial Fibrillation and Flutter | Field IDs 131350, 131351 |
| Cancer | Curated from Field IDs 40009 and 40005 |
| Chronic Renal Failure | Field IDs 132032, 132033 |
| COPD | Field IDs 42016, 42017 |
| Dementia | Field IDs 42018, 42019 |
| Depression | Field IDs 130892, 130893, 130894, 130895, 130896, 130897 |
| Diabetes | Field IDs 130706, 130707, 130708, 130709, 130710, 130711, 130712, 130713 |
| Heart Failure | Field IDs 131354, 131355 |
| Myocardial Infarction | Field IDs 42000, 42001 |
| Obesity | Field IDs 130792, 130393 |
| Osteoarthritis | Field IDs 42040, 41262, 41202, 41263  Secondary care: ICD10 Codes M150-154, M158-M167, M169-M175, M179-M185, M189-M192, M198 and ICD9 Codes 715, 7150, 7151, 7152, 7153, 7158, 7159. Primary care Read V2 Code N05.. and Read CTV3 Code XE1DV |
| Peripheral Arterial Disease | Field IDs 131388, 131389, 131390, 131391, 131384, 131385, 131382, 131383 |
| Primary Hypertension | Field IDs 131286, 131287 |
| Stroke | Field IDs 42006, 42007 |
| Thyroid Disease | Field IDs 130720, 130721, 130722, 130723, 130692, 130693, 130694, 130695, 130696, 130697, 130698, 130699, 130700, 130701, 130702, 130703, 130704, 130705 |
| Sex | Field ID 31 |
| Month of birth | Field ID 52 |
| Year of birth | Field ID 34 |
| Ethnicity | Field ID 2100  White defined as British White, Irish White or Any other white background. Non-white defined as any other stated ethnicity. |

Further details relating to these codes are provided by UK Biobank via the online data showcase: <https://biobank.ndph.ox.ac.uk/showcase/>

**Supplemental Table 2: Timing of comorbidity diagnoses in relation to HF diagnosis in participants with at least 5 years follow-up or death in this period**

|  | **Time in relation to HF (years)** | |
| --- | --- | --- |
| **Comorbidity** | **Primary analysis** | **Sensitivity analysis** |
| Osteoarthritis | -6.9 (-12.9 to -1.5) | -5.4 (-11.9 to 0.0) |
| Depression | -4.6 (-14.4 to 0.0) | -2.5 (-11.2 to 0.4) |
| Obesity | -3.8 (- 11.2 to 0.0) | -3.0 (-10.7 to 0.9) |
| Cancer | -3.8 (-11.6 to 0.0) | -2.0 (-9.4 to 1.1) |
| Diabetes | -3.4 (-10.3 to 0.0) | -2.1 (-9.5 to 1.5) |
| Primary Hypertension | -2.6 (-9.8 to 0.0) | -1.6 (-8.8 to 0.5) |
| Thyroid Disease | -1.7 (-10.2 to 0.7) | -0.6 (-9.4 to 2.3) |
| Asthma | -1.3 (-7.4 to 0.6) | -0.5 (-6.8 to 3.3) |
| Anaemia | -0.3 (-6.1 to 0.9) | -0.1 (-5.2 to 1.8) |
| Atrial Fibrillation & Flutter | -0.1 (-3.9 to 0.0) | 0.0 (-3.2 to 0.3) |
| Chronic Renal Failure | 0.0 (-6.4 to 2.0) | 0.0 (-4.5 to 3.5) |
| Peripheral Arterial Disease | 0.0 (-4.8 to 1.4) | 0.0 (-4.5 to 2.5) |
| COPD | 0.0 (-3.6 to 1.5) | 0.0 (-2.7 to 3.4) |
| Myocardial Infarction | 0.0 (-2.4 to 0.0) | 0.0 (-1.6 to 0.1) |
| Stroke | 0.0 (-3.2 to 2.9) | 0.0 (-2.0 to 4.7) |
| Dementia | 0.7 (-0.1 to 4.8) | 0.6 (-0.1 to 5.5) |

Data presented as median (first quartile to third quartile).

**Supplemental Table 3:** **Timing of comorbidity diagnoses in relation to HF diagnosis stratified by sex**

|  | **Time in relation to HF (years)** | |
| --- | --- | --- |
| **Comorbidity** | **Male** | **Female** |
| Osteoarthritis | -6.2 (-12.5 to -0.9) | -7.5 (-13.4 to -2.1) |
| Depression | -3.6 (-13.4 to 0.1) | -6.4 (-15.7 to 0.0) |
| Obesity | -3.0 (-10.5 to 0.2) | -5.3 (12.2 to 0.0) |
| Cancer | -2.6 (-9.3 to 0.8) | -7.1 (-15.6 to -0.8) |
| Diabetes | -3.3 (-10.0 to 0.0) | -3.4 (-10.9 to 0.0) |
| Primary Hypertension | -2.0 (-9.5 to 0.0) | -3.4 (-10.4 to 0.0) |
| Thyroid Disease | 0.0 (-5.8 (to 2.3) | -4.1 (-13.7 to 0.0) |
| Asthma | -0.3 (-5.6 to 2.0) | -2.9 (-10.1 to 0.0) |
| Anaemia | 0.0 (-4.5 to 2.1) | -1.5 (-8.6 to 0.2) |
| Atrial Fibrillation & Flutter | -0.1 (-3.9 to 0.0) | -0.1 (-3.8 to 0.0) |
| Chronic Renal Failure | 0.0 (-5.7 to 2.4) | -0.5 (-7.3 to 1.3) |
| Peripheral Arterial Disease | 0.0 (-4.8 to 1.9) | -0.1 (-4.9 to 0.6) |
| COPD | 0.0 (-2.7 to 2.1) | -0.3 (-4.8 to 0.6) |
| Myocardial Infarction | 0.0 (-3.0 to 0.0) | 0.0 (-1.6 to 0.0) |
| Stroke | 0.0 (-3.3 to 3.9) | 0.0 (-2.9 to 1.7) |
| Dementia | 0.7 (-0.1 to 4.6) | 0.0 (-0.2 to 5.1) |

Data presented as median (first quartile to third quartile)

**Supplemental Table 4:** **Timing of comorbidity diagnoses in relation to HF diagnosis stratified by age at HF diagnosis**

|  | **Time in relation to HF (years)** | | | | |
| --- | --- | --- | --- | --- | --- |
| **Comorbidity** | **HF aged <50** | **HF aged 50-60** | **HF aged 60-70** | **HF aged 70-80** | **HF aged 80-90** |
| Osteoarthritis | 7.0 (1.5 to 12.2) | 0.7 (-4.7 to 7.9) | -3.8 (-9.6 to 0.3) | -8.8 (-14.4 to -3.8) | -11.7 (-16.3 to -6.8) |
| Depression | 0.5 (-5.0 to 7.0) | -1.1 (-8.5 to 3.7) | -4.3 (-13.4 to 0.1) | -6.5 (-17.2 to -0.1) | -7.6 (-18.6 to -0.7) |
| Obesity | 0.8 (-2.9 to 9.1) | 0.0 (-5.0 to 6.0) | -3.1 (-10.2 to 0.4) | -5.7 (-13.0 to 0.0) | -8.3 (-14.8 to -2.1) |
| Cancer | 0.6 (-7.8 to 11.7) | 1.9 (-4.4 to 9.0) | -1.2 (-8.3 to 2.9) | -5.8 (-13.0 to -0.9) | -10.3 (-16.9 to -4.0) |
| Diabetes | 5.5 (0.0 to 13.6) | 0.0 (-4.2 to 6.4) | -2.3 (-8.6 to 0.5) | -5.8 (-12.4 to -0.3) | -7.9 (-14.8 to -2.3) |
| Primary Hypertension | 1.4 (-0.1 to 10.0) | 0.0 (-3.0 to 4.1) | -0.4 (-7.4 to 0.9) | -5.3 (-12.3 to -0.1) | -8.1 (-13.4 to -2.2) |
| Thyroid Disease | 8.2 (0.5 to 14.1) | 1.3 (-2.8 to 6.9) | -0.4 (-7.8 to 1.6) | -4.0 (-13.8 to 0.0) | -3.9 (-14.2 to -0.5) |
| Asthma | 1.7 (-1.9 to 13.0) | 0.0 (-1.8 to 10.9) | -0.1 (-6.2 to 2.3) | -3.1 (-8.6 to 0.0) | -6.8 (-12.1 to -0.5) |
| Anaemia | 7.9 (0.0 to 16.2) | 0.6 (-1.9 to 8.1) | 0.0 (-4.8 to 2.9) | -1.0 (-6.9 to 0.1) | -2.6 (-8.9 to 0.0) |
| Atrial Fibrillation & Flutter | 4.9 (0.0 to 13.1) | 0.0 (-0.3 to 6.5) | 0.0 (-2.2 to 0.1) | -0.4 (-5.3 to 0.0) | -2.0 (-7.7 to 0.0) |
| Chronic Renal Failure | 8.3 (0.4 to 15.8) | 4.8 (0.0 to 10.1) | 0.3 (-2.9 to 4.2) | -1.4 (-8.2 to 0.2) | -5.2 (-12.6 to 0.0) |
| Peripheral Arterial Disease | 2.9 (0.0 to 14.5) | 2.7 (-0.7 to 9.2) | 0.0 (-2.3 to 4.0) | -1.0 (-6.6 to 0.0) | -2.0 (-7.5 to 0.0) |
| COPD | 8.0 (1.5 to 14.2) | 4.8 (0.0 to 11.4) | 0.0 (-2.1 to 4.2) | -0.6 (-4.8 to 0.0) | -1.8 (-6.3 to 0.0) |
| Myocardial Infarction | 0.0 (-0.4 to 3.5) | 0.0 (-0.6 to 0.0) | 0.0 (-1.6 to 0.1) | 0.0 (-3.4 to 0.0) | -1.3 (-6.4 to 0.0) |
| Stroke | 8.3 (4.6 to 16.0) | 4.7 (0.0 to 11.3) | 0.2 (-0.9 to 5.8) | -0.2 (-4.6 to 0.7) | -0.8 (-7.3 to 0.0) |
| Dementia | 13.2 (9.2 to 18.6) | 15.9 (10.4 to 18.8) | 6.0 (0.7 to 10.1) | 0.3 (-0.3 to 2.9) | -0.1 (-2.0 to 0.0) |

Data presented as median (first quartile to third quartile)

**Supplemental Figure 1:** **Timing of comorbidity diagnoses in relation to HF diagnosis stratified by sex**


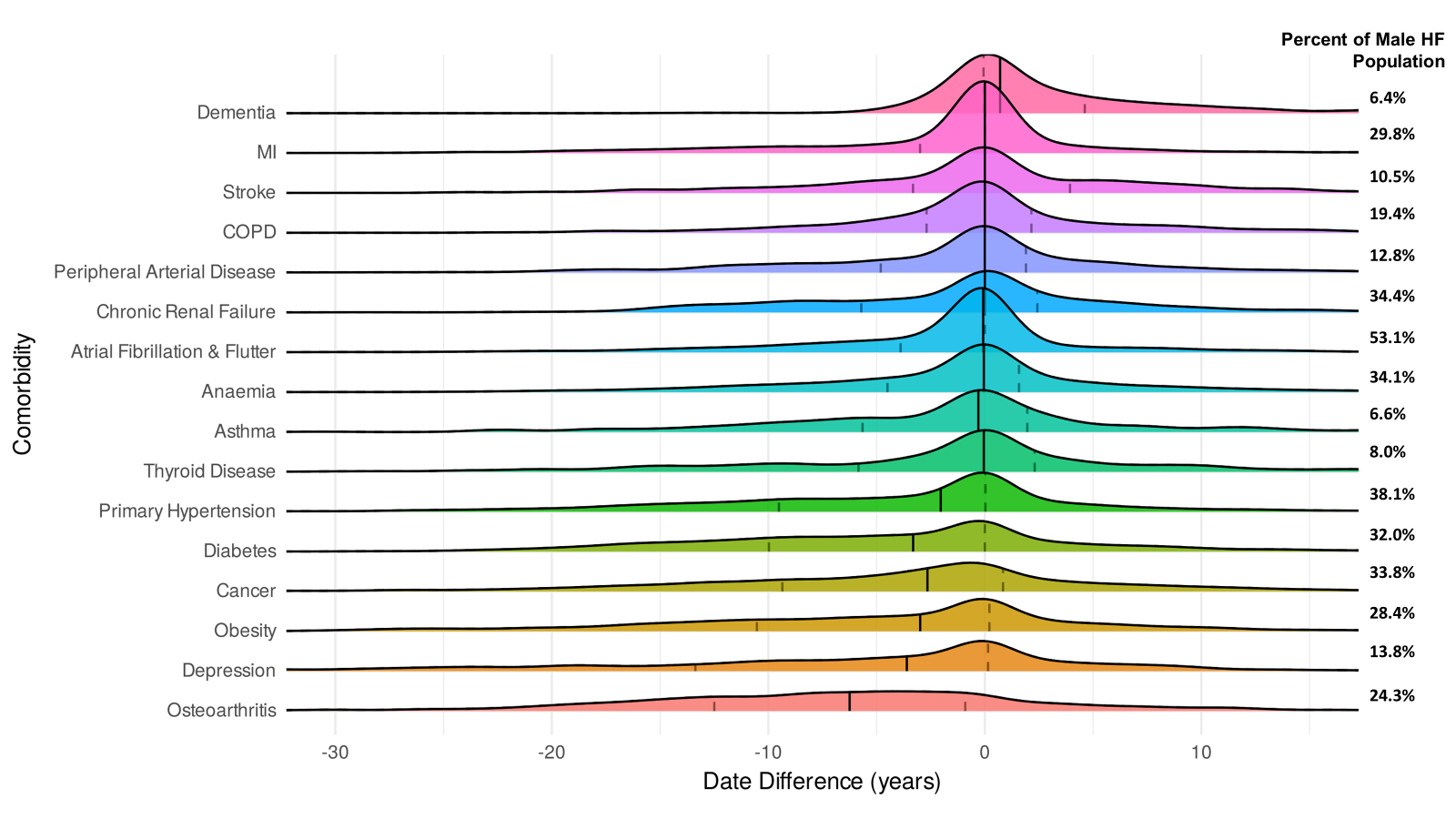


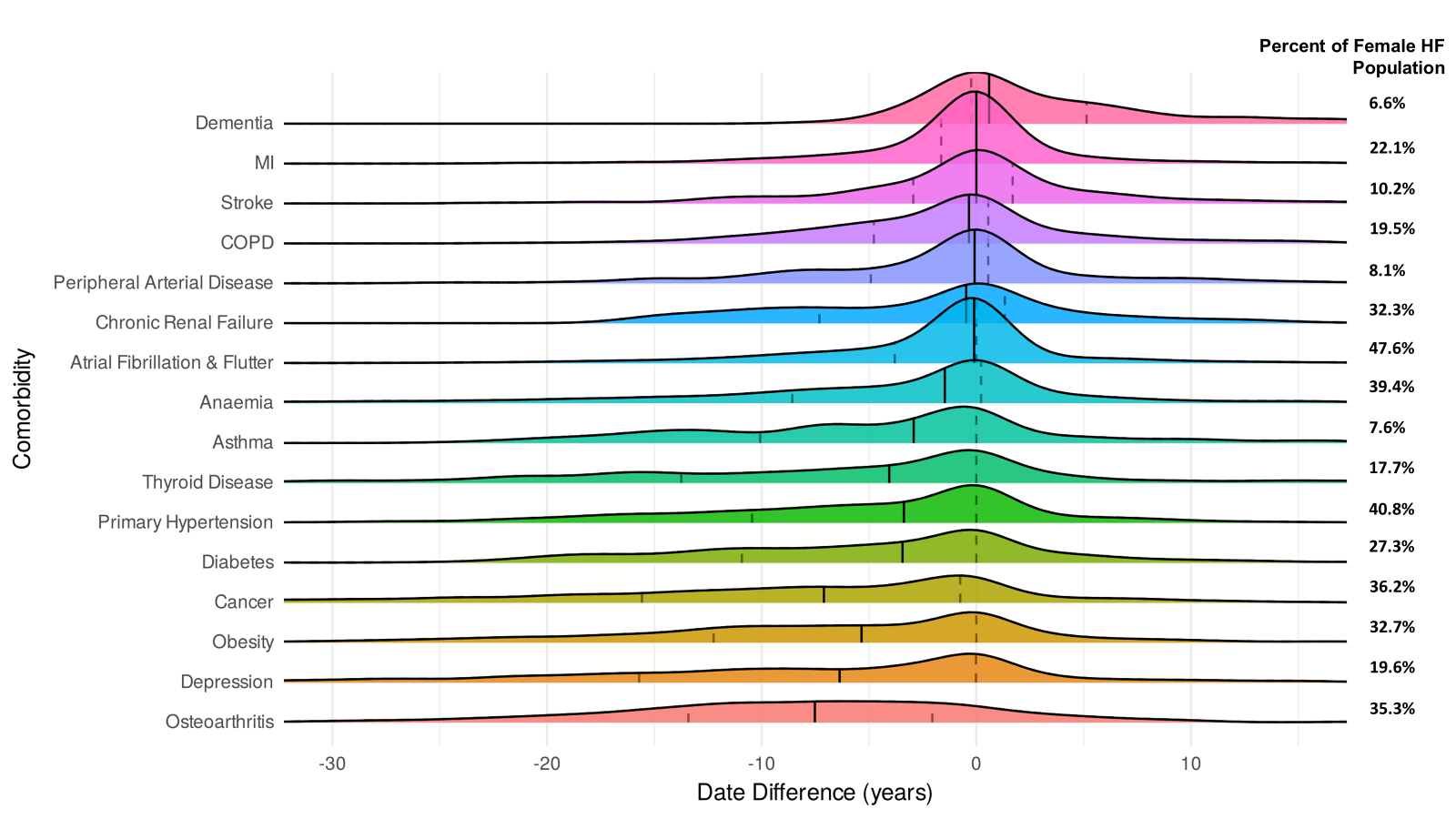


Ridgeline plots illustrating the chronological distribution of comorbidity diagnoses in relation to heart failure (HF) diagnosis in men and women. The amplitude of Ridgelines does not relate to comorbidity prevalence, the data for which are noted to the right of the plot for reference. MI - myocardial infarction; COPD – chronic obstructive pulmonary disease.

**Supplemental Figure 2:** **Timing of comorbidity diagnoses in relation to HF diagnosis stratified by age at HF diagnosis**


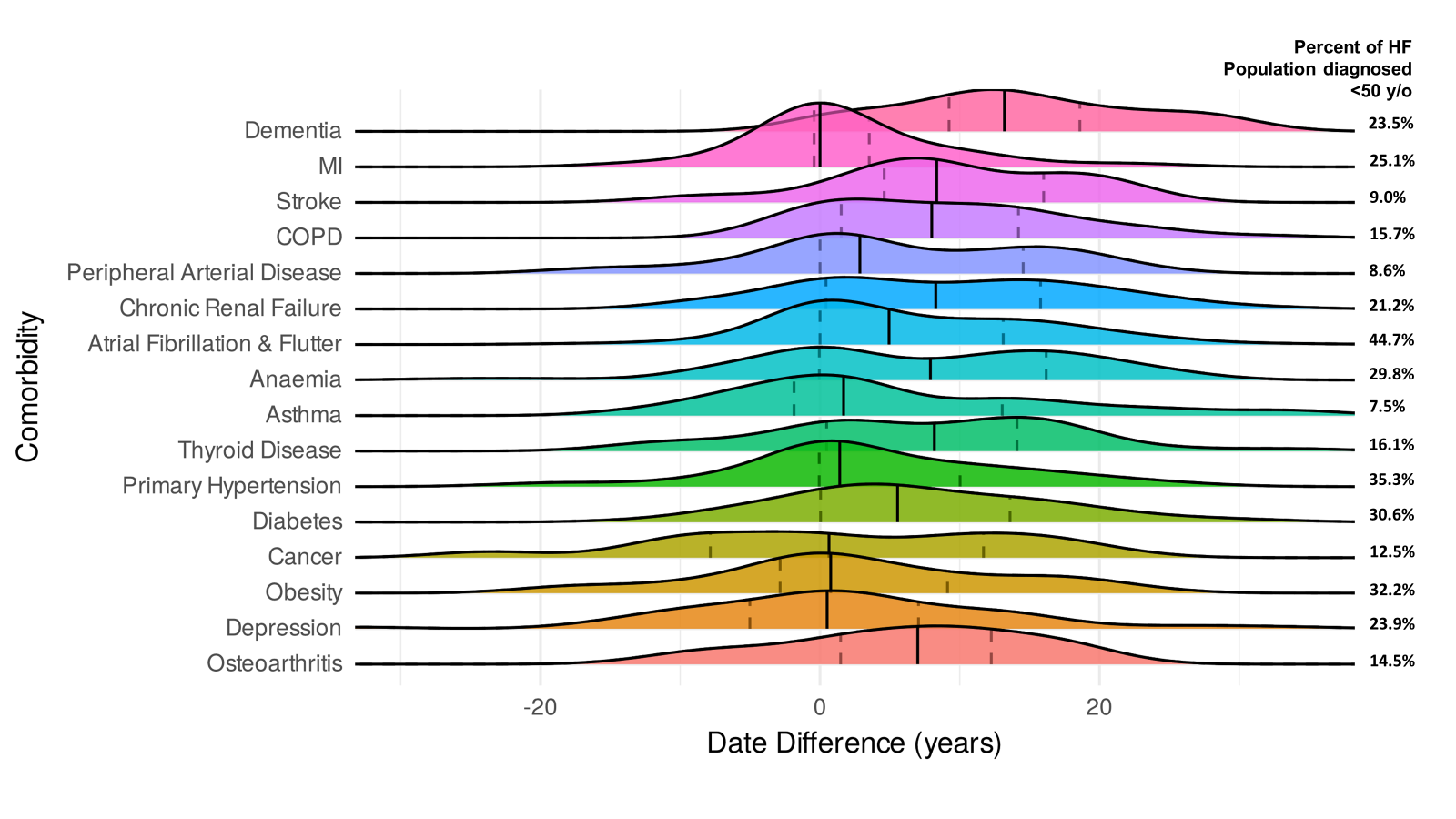


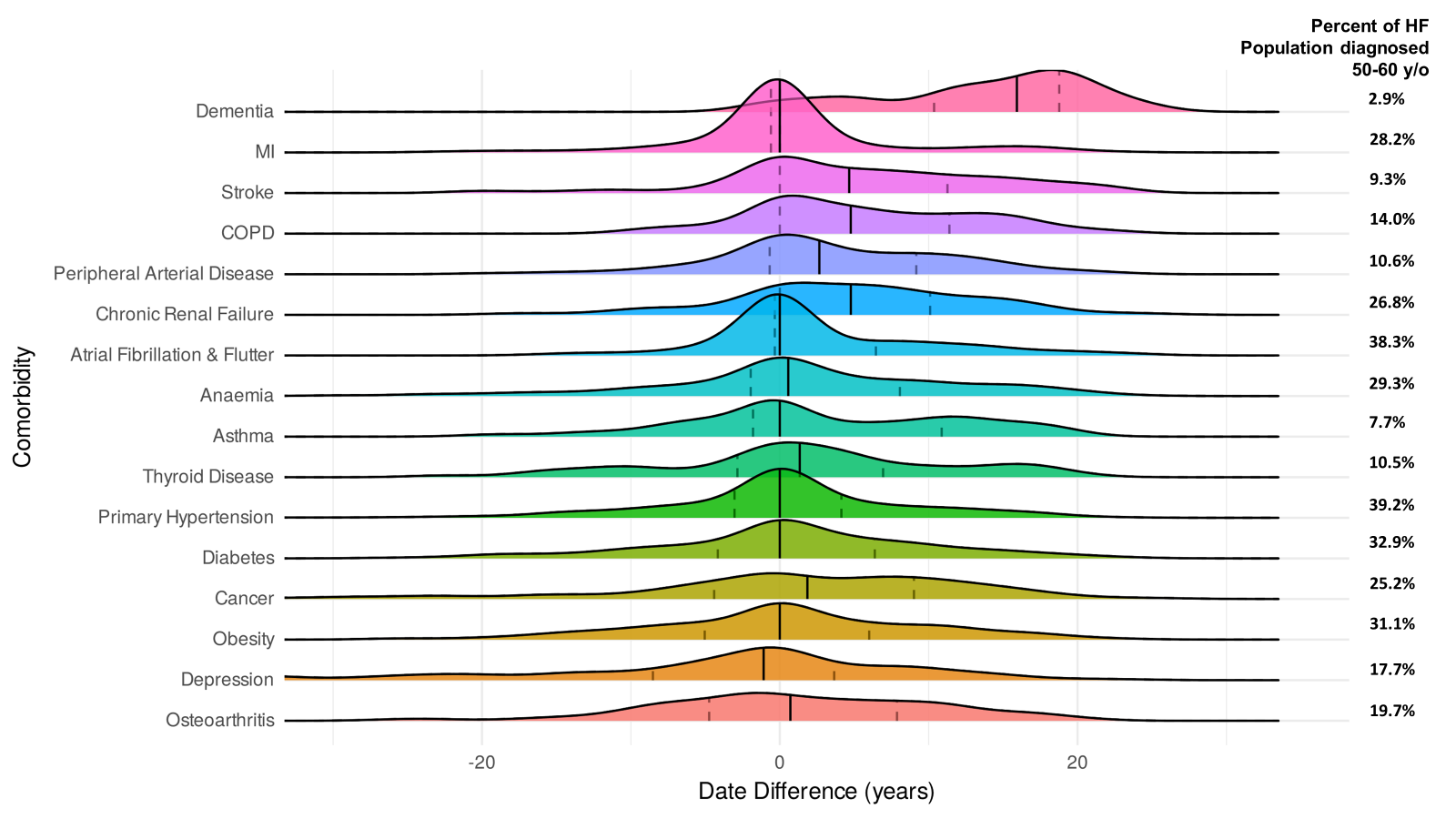


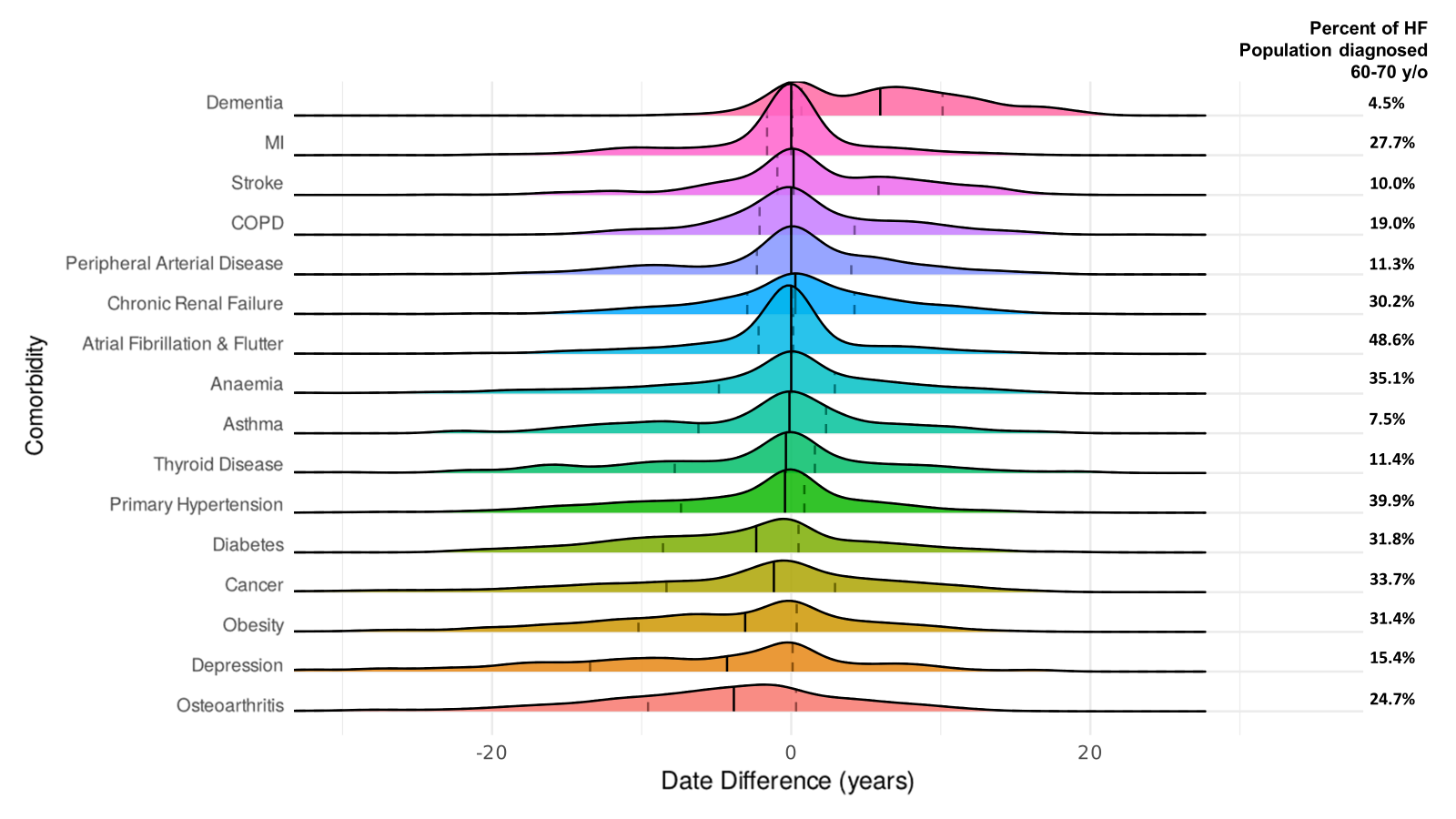


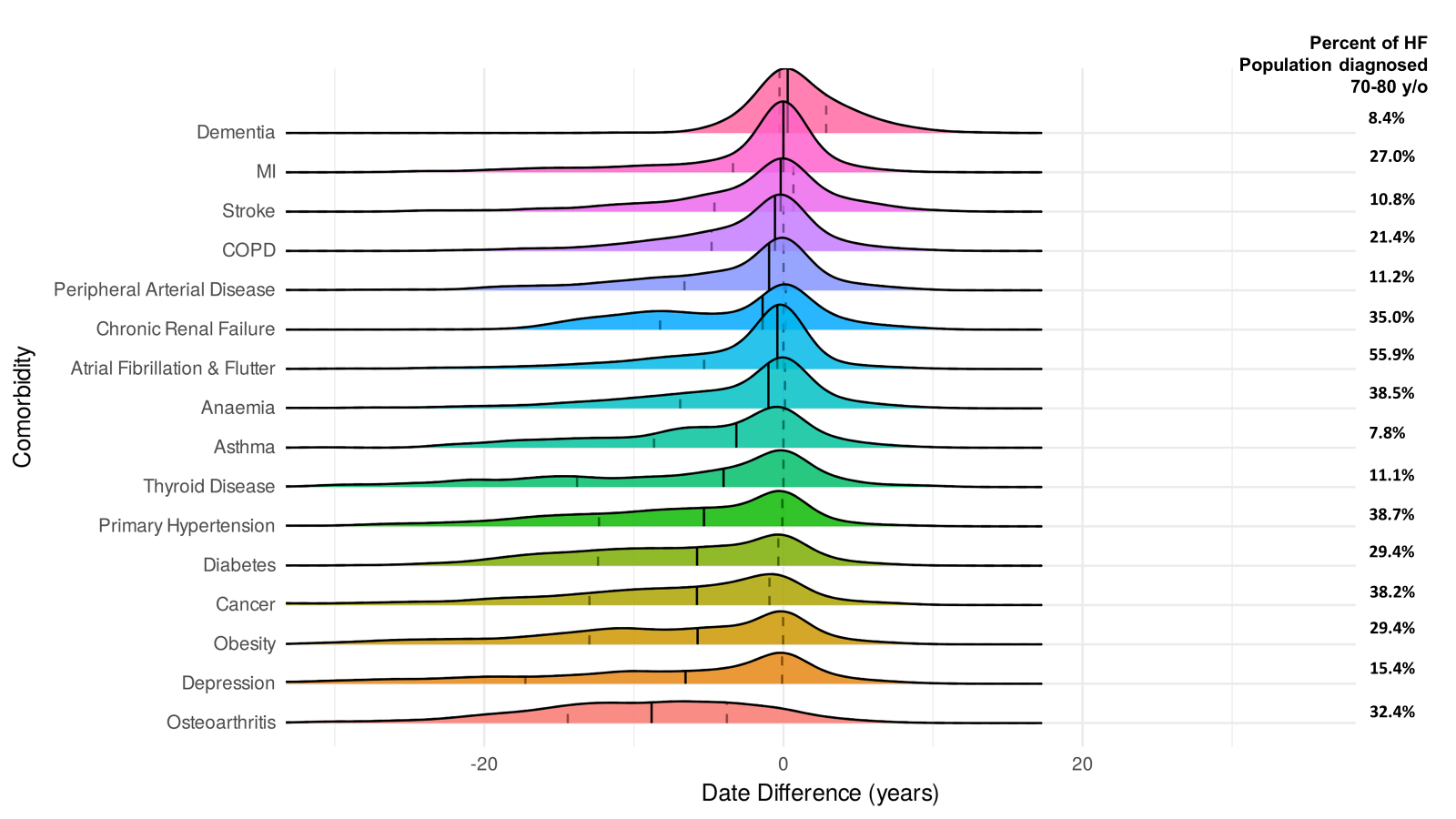


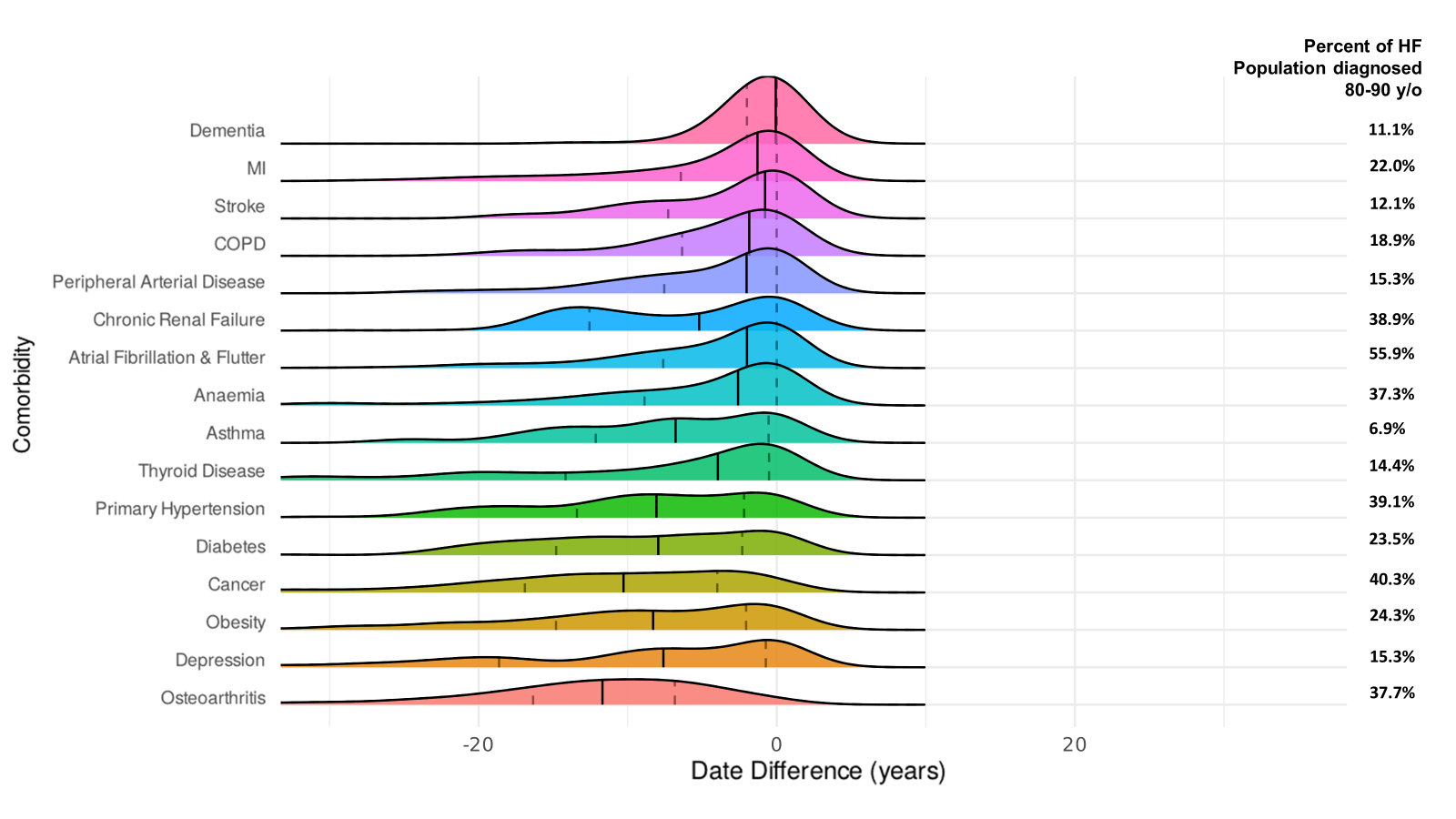


Ridgeline plots illustrating the chronological distribution of comorbidity diagnoses in relation to heart failure (HF) diagnosis for each stratum of age at HF diagnosis. The amplitude of Ridgelines does not relate to comorbidity prevalence, the data for which are noted to the right of the plot for reference. MI - myocardial infarction; COPD – chronic obstructive pulmonary disease.
